# Immune Responses Following BCG Immunization of Infants in Uganda and United Kingdom Are Similar for Purified Protein Derivative but Differ for Secretory Proteins of *Mycobacterium tuberculosis*

**DOI:** 10.3389/fimmu.2021.637114

**Published:** 2021-03-19

**Authors:** Patrice A. Mawa, Mateusz Hasso-Agopsowicz, Lawrence Lubyayi, Grace Nabakooza, Marjorie Nakibuule, Rose Blitz, Li Dun, Abha Govind, Pontiano Kaleebu, Emily L. Webb, Alison M. Elliott, Hazel M. Dockrell, Stephen Cose, Steven G. Smith

**Affiliations:** ^1^Immunomodulation and Vaccines Programme, Medical Research Council-Uganda Virus Research Institute and London School of Hygiene & Tropical Medicine Uganda Research Unit, Entebbe, Uganda; ^2^Department of Immunology, Uganda Virus Research Institute, Entebbe, Uganda; ^3^Department of Infection Biology, London School of Hygiene and Tropical Medicine, London, United Kingdom; ^4^Department of Epidemiology and Biostatistics, School of Public Health, University of the Witwatersrand, Johannesburg, South Africa; ^5^Fetal Medicine Unit, Gynaecology and Obstetrics Department, North Middlesex University Hospital National Health Service Trust, London, United Kingdom; ^6^Medical Research Council Tropical Epidemiology Group, Department of Infectious Disease Epidemiology, London School of Hygiene and Tropical Medicine, London, United Kingdom; ^7^Department of Clinical Research, London School of Hygiene and Tropical Medicine, London, United Kingdom

**Keywords:** BCG vaccine, cytokine responses, chemokine responses, mycobacterial antigens, latent *Mycobacterium tuberculosis* infection

## Abstract

**Introduction:** The immunogenicity of BCG vaccination in infants differs between populations. We hypothesized that prenatal exposure to mycobacterial antigens might explain the differences in immune responses to BCG seen in other studies of infants in Africa and the United Kingdom (UK) and we explored this in birth cohorts in Uganda and the UK.

**Materials and Methods:** Blood samples were obtained from BCG-immunized infants of mothers with (*n* = 110) and without (*n* = 121) latent *Mycobacterium tuberculosis* infection (LTBI) in Uganda and BCG-immunized infants of mothers without LTBI (*n* = 25) in the UK at 10 and 52 weeks after birth. Cytokine and chemokine responses to PPD were measured to assess responses to BCG immunization, and to ESAT6/CFP10 to assess exposure to or infection with *M. tuberculosis* or non-tuberculous mycobacteria (NTM) in 6-day whole blood culture supernatants by a 17-plex Luminex assay. Median responses were compared between Ugandan infants (together, and separated by maternal LTBI status) and UK infants.

**Results:** The IFN-γ response to BCG vaccination was similar between Ugandan and UK infants at 10 and 52 weeks. At week 52, TNF production was marginally higher in Ugandan infants, but after adjusting for multiple comparisons this difference was not significant. At weeks 10 and 52, stimulation of blood with ESAT6/CFP10 produced significantly higher IFN-γ, TNF, IL-12p40, IL-1α, IL-1β, IL-1Ra, IP-10, MIP-1α, MIP-1β, and GM-CSF in Ugandan compared to UK infants. Stimulation of blood with ESAT6/CFP10 produced significantly higher amounts of IL-8 (*p* = 0.0001), IL-10 (*p* = 0.0022), and IL-13 (*p* = 0.0020) in the UK than in Ugandan infants of mothers without LTBI at week 10, but not at week 52.

**Conclusions:** Immune responses to mycobacterial antigens following BCG immunization are similar for PPD, but differ for ESAT6/CFP10, between infants in Uganda and the UK. Neither maternal LTBI nor infant exposure to or infection with mycobacteria impacts the response to BCG. The observed global differences in immune response to BCG immunization are likely to be due to other causes.

## Introduction

The protective efficacy of Bacille Calmette-Guerin against tuberculosis (TB) varies geographically, ranging from 0 to 80% (2–4), with lower protection demonstrated in tropical countries (5). Factors including exposure to non-tuberculous mycobacteria (NTM) (4, 5) and other common infections in the tropics (6) may alter the efficacy of BCG immunization.

BCG immunization of infants induces a measurable immune response to mycobacterial antigens, such as purified protein derivative (6) of *Mycobacterium tuberculosis*, with infants shown to generate cytokine-expressing T cell responses of the same magnitude as adults, (7) characteristically with a T helper (Th) 1 bias (8). Various studies have demonstrated T-cell responses to mycobacterial antigens following BCG immunization of infants (9–12). However, studies in Uganda, Malawi, The Gambia, Indonesia and the UK (9, 11, 13–16) have shown different patterns of response to BCG immunization in infants, with Th1 responses predominant among infants in the UK, contrasting with Th2 responses seen among infants in Malawi (13). Responses among infants in Malawi and The Gambia were partially explained by the season of birth (11, 14).

The profile of immune response to BCG immunization seen in infants from Malawi may be similar to other settings in sub-Saharan Africa where the protective efficacy of BCG is low. We aimed to explore whether prenatal exposure to mycobacterial antigens explained the Africa-UK differences to any extent using samples obtained from a study that investigated the impact of maternal latent *M. tuberculosis* infection (LTBI) on the infant response to BCG immunization (17). In murine studies, mycobacterial antigens have been shown to cross the placenta following gestational treatment with mycobacterial antigens (18). Our proposition is therefore that maternal LTBI might lead to exposure to mycobacterial antigens *in utero* resulting in sensitization (19), or tolerance (19, 20) in the unborn babies. Passive transfer of maternal anti-mycobacterial antibodies or maternal anti-idiotype antibodies (mimicking antigen) have been proposed as an alternative mechanism (21). The maternal and placental immunological environment could also be affected non-specifically by maternal LTBI, with impact on neonatal and fetal immune responses (22).

We analyzed and compared responses between infants of mothers with and without LTBI in Uganda and infants of mothers without LTBI in the UK and hypothesized that the responses to BCG immunization would differ between infants in Uganda and the UK. We also anticipated that responses in Ugandan infants of mothers without LTBI would be more similar to responses in UK infants than responses in Ugandan infants of mothers with LTBI.

## Materials and Methods

### Study Design and Participants

Between June 2014 and October 2016, healthy Ugandan mothers and their infants were recruited at Entebbe General Hospital as part of a larger study to investigate the impact of maternal LTBI on the infant response to BCG immunization (17).

To be included in the study, women had to give consent to participate, have a normal singleton pregnancy, have an uncomplicated delivery of a neonate >2,500 g, be residing in Entebbe municipality or Katabi sub-county, and be HIV negative. Participants among whom cord blood was not obtained, delivery was not normal, the mother was unwilling to undergo a repeat HIV test or found to be HIV-positive on repeat testing, the mother had indeterminate LTBI status, the neonate was unwell as judged by the midwife, or the neonate presented with significant congenital abnormalities likely to impair the child's general health and development were excluded. All vaccines recommended by the Expanded Programme (EPI) on Immunization were administered to the enrolled Ugandan infants and these included oral poliovirus vaccine (OPV) at birth, diphtheria-tetanus toxoids and pertussis (DTP), *Haemophilus influenzae* type B (HiB), hepatitis B (HBV) and OPV at 6, 10, and 14 weeks of age, and measles at 9 months of age.

Between March 2015 and August 2017, infants in the UK were recruited at North Middlesex Hospital, London if their mothers were willing to participate, had a singleton pregnancy, had an uncomplicated delivery of a neonate >2,500 g and were LTBI-negative as determined by the T-SPOT.TB assay (Oxford Immunotec). Infant vaccines administered in the UK were the 6-in-1 vaccine (diphtheria, hepatitis B, HiB, polio, tetanus, pertussis, rotavirus vaccine and meningococcal (Men) B at 8 weeks, 6-in-1 vaccine (2nd dose), pneumococcal conjugate vaccine (PCV) (1st dose) vaccine and rotavirus vaccine (2nd dose) at 12 weeks, 6-in-1 vaccine (3rd dose) and MenB (2nd dose) at 16 weeks and Hib/MenC (1st dose), MMR (measles, mumps and rubella) (1st dose), PCV vaccine (2nd dose) and MenB (3rd dose) at 52 weeks.

All Ugandan and UK infants were BCG-immunized at birth or within the first week of life with BCG Danish, Statens Serum Institut (SSI). Infants in Uganda were randomly assigned in a 1:1 ratio (stratified by maternal LTBI status) to two sampling strategies to reduce the blood-sampling burden on individual infants. This resulted in only half the Ugandan infants sampled at week 10. Peripheral venous blood samples were collected from infants of mothers with and without LTBI in Uganda at 10 and 52 weeks after BCG immunization. In the UK, maternal LTBI was not determined prior to collecting a sample from the infants at 10 and 52 weeks. Therefore, all samples were collected regardless of maternal LTBI status, but only samples from infants of mothers without LTBI were used for analysis.

### Tests for Latent TB Infection

In Uganda, women were examined for LTBI at ~1 week post-delivery using the tuberculin skin test (TST) (PPD RT23 SSI, Copenhagen, Denmark) and T-SPOT.TB assay (Oxford Immunotec, Abingdon, UK). The TST was performed in the mothers after bleeding for the T-SPOT.TB assay and was read 48–72 h later, and defined as positive if ≥10 mm in diameter. Women positive on both tests were considered LTBI-positive; those negative on both tests were considered LTBI-negative; those with indeterminate and inconsistent results were excluded. LTBI-positive mothers were examined for active tuberculosis by symptoms, sputum examination (if available), and chest x-ray and there were no cases of active TB disease detected in mothers and their infants during the study period, and data available on the subjects has been reported (17).

In the UK, a 5 ml blood sample was obtained from women at the infant's 10-week appointment and tested for LTBI using the T-SPOT.TB assay. If the mother tested positive, infants were excluded from the study and the clinical team at North Middlesex Hospital were informed of the result. TST testing was not available in the UK.

### Whole Blood Assays for Cytokine and Chemokine Responses

Identical standard operating procedures were used in Uganda and the UK since the assays were performed in the different laboratories. Reagents from the same manufacturers were used. Venous blood was diluted 1/5 in RPMI 1640 (Invitrogen) supplemented with 2 mM L-glutamine (Invitrogen) and cultured at 37°C in 5% CO_2_ for 6 days in 96-well U-bottomed plates. Duplicate wells were incubated with medium alone (negative control), PPD (Statens Serum Institut, catalog # RT50; 10 μg/mL), and with a combination of *M. tuberculosis*-specific antigens [6 kDa Early Secreted Antigen of *M. tuberculosis* (ESAT-6) and 10 kDa culture filtrate protein (CFP10)] (BEI Resources, catalog #s NR14868 and NR-49425; each at 5μg/mL).

After 6 days, plates were centrifuged at 400 g for 5 min. Supernatants were removed from duplicate wells, pooled, and stored at −80°C prior to analysis. Thawed supernatants were randomized across plates and analyzed using the multiplex bead array using the human cytokine/chemokine Milliplex^TM^ MAP 17-plex pre-mixed kit (Merck Millipore), following the manufacturer's instructions. The pre-mixed bead set included interleukin (IL)-1α, IL-1β, IL-1Ra, IL-2, IL-5, IL-8, IL-10, IL-12p40, IL-13, IL-17A, interferon (IFN)-γ, IFN-γ-inducible protein (IP)-10, monocyte chemotactic protein (MCP)-1, macrophage inflammatory protein (MIP)-1α, MIP-1β, tumor necrosis factor (TNF), and granulocyte macrophage colony-stimulating factor (GM-CSF). Reference samples were included at both sites to ensure data comparability. Data were acquired using the Biorad Luminex® 200 system and Bioplex Manager Software version 6.1 (Biorad).

### Statistical Methods

We conducted analyses to compare immune responses to mycobacterial antigens in Uganda and UK infants using blood samples collected at 10 and 52 weeks following BCG immunization. For the larger study (17), in Uganda, we aimed to recruit 150 women with LTBI and 150 without, to give 80% power to detect a difference of 0.35log10 (assuming a standard deviation of 0.9log10) in infant cytokine response at 52 weeks between the two groups, and a difference of 0.5log10 at other time points (with 75 infants in each group). In the UK, we aimed to recruit a comparison group of 150 women.

The values of cytokine and chemokine response from unstimulated samples were subtracted from values from antigen-stimulated samples. Values <3.2 pg/ml were assigned as 3.2 pg/ml. Values above the upper detection limit were assigned 11,000 pg/ml.

Baseline characteristics of participants were summarized by LTBI exposure status (for infants in Uganda) and by country using percentages, medians and ranges. Mann Whitney and chi-squared tests were used to compare baseline characteristics between Ugandan and UK infants. Cytokine and chemokine responses were compared by LTBI exposure status, country, and each time point using the Mann-Whitney U test. Dot plots were used to visualize distributions of log-transformed cytokine and chemokine responses at weeks 10 and 52; log-transformed responses were preferred because they offered clearer visual comparisons as compared to the raw values. These are presented as Log_10_ analyte concentration measured in pg/ml. A significance level of 0.003 (0.05/17) was used, considering a Bonferroni correction for multiple comparisons.

Data analysis and presentation were conducted using Stata 15.1 (College Station, Texas, USA) and PRISM v8.11 (224) (GraphPad software, Inc., La Jolla, CA, USA).

### Ethics Approval Statement

Ethics approval was given by the Uganda Virus Research Institute Research Ethics Committee (reference GC/127/13/09/16 and GC/127/16/03/434), Uganda National Council for Science and Technology (reference HS 1526) and London School of Hygiene & Tropical Medicine (reference 7104 for the Uganda study, 8720-1 for the UK study) and 15/LO/0048 for the NHS in the UK study. All mothers gave written informed consent for their own and their baby's participation in the study.

## Results

### Characteristics of Study Participants

Baseline characteristics of participants are shown in Table 1. Mothers from the UK were older on average than those from Uganda (*p* < 0.001), but median birth weight (*p* = 0.099) and sex (*p* = 0.776) distributions were similar across groups. Other details about the subjects in this study have been reported as part of the larger study, including inclusion of those who were HIV-negative and a lack of difference in the prevalence of helminth infections between LTBI positive and negative groups in Uganda (17).

**Table 1:** Characteristics of study participants.

	**UG LTBI– (*n* = 121)**	**UG LTBI+ (*n* = 110)**	**UK (*n* = 25)**	***p*-value**
Mother's age (years), median (range)	23 (17–37)	25 (17–42)	34 (19–43)	<0.001
Birth weight (kg), median (range)	3.2 (2.5–4.5)	3.2 (2.5–4.4)	3.35 (2.73–4.3)	0.099
Sex of the baby, male *n* (%)	63 (52%)	64 (58%)	13 (52%)	0.776

*P-values are from the Mann Whitney-test for comparisons of mother's age and birth weight and from the chi-squared-test for comparison of sex proportions between infants from Uganda (overall) and those from the UK*.

A general comparison of median cytokine responses between all infants in Uganda and the UK regardless of maternal LTBI status in Uganda was performed. These responses were further analyzed to examine the impact of maternal LTBI. Due to loss of some study subjects to follow up, responses to PPD were examined at week 10 for 51 infants of mothers with LTBI (UG+) and 57 infants of mothers without LTBI (UG–) in Uganda and 25 infants in the UK (UK); and at week 52 for 95 infants of mothers with LTBI (UG+) and 110 infants of mothers without LTBI (UG–) in Uganda and 12 infants in the UK (UK).

For ESAT6/CFP10, responses were examined at week 10 for 39 infants of mothers with (UG+) and 41 infants of mothers without LTBI (UG–) in Uganda and 25 infants in the UK (UK); and at week 52 for 95 infants of mothers with LTBI (UG+) and 109 infants of mothers without LTBI (UG–) in Uganda and 12 infants in the UK (UK).

### Immune Response to PPD Stimulation

Cytokine and chemokine responses following stimulation of infant whole blood with PPD were measured to assess responses induced by BCG immunization.

At weeks 10 and 52, there were no differences in median IFN-γ production in response to PPD stimulation of whole blood between infants in Uganda and the UK (*p* = 0.2638, *p* = 0.8909, respectively). At week 52, median TNF production was marginally higher in Ugandan infants, but after correction for multiple testing this was not significant (*p* = 0.0078, Table 2).

**Table 2:** Median responses to PPD in infants from Uganda and United Kingdom at 10 and 52 weeks following BCG immunization.

**Cytokine/ Chemokine (pg/ml)**	**10 weeks after BCG**			**52 weeks after BCG**		
	**Uganda(*n* = 108)**	**United Kingdom(*n* = 25)**	***p*-value**	**Uganda(*n* = 205)**	**United Kingdom (*n* = 12)**	***p*-value**
IFN-γ	337.5	326.8	0.2638	264.7	265.1	0.8909
TNF	126	59.7	0.2636	69.26	36.04	0.0078
IL-2	5.51	25.5	**0.0001**	14.86	26.34	0.2908
IL-12p40	2.85	5.97	0.1256	2.395	4.370	0.5677
IL-1α	60.44	94.9	0.4134	10.21	27.25	0.2838
IL-1β	58.96	7.8	**<0.0001**	22.82	3.535	0.0090
IL-1Ra	180.30	164.6	0.4201	105.9	102.0	0.5018
IL-17A	16.87	22.2	0.1909	14.57	13.37	0.8108
IL-5	36.75	37.2	0.3394	27.92	39.21	0.2417
IL-13	163.8	319.1	0.0595	119.2	138.5	0.3788
IL-10	0.00	7.6	**<0.0001**	1.64	5.64	0.2087
IL-8	10,159	10605.3	0.0188	10,003	10,609	0.0115
IP-10	10,409	10,877	**<0.0001**	9,846	10,882	**0.0001**
MCP-1	4,294	3,525	0.3529	6,133	2,535	0.0104
MIP-1α	230.5	212.4	0.5594	47.49	57.10	0.2915
MIP-1β	268.9	366.6	0.2972	43.85	126.8	**0.0013**
GM-CSF	413.8	432.9	0.8493	122.5	274.5	0.0306

*P-values are from the Mann Whitney-test for comparison of responses between infants from Uganda and those from the UK. Bold-faced values represent statistically significant p-values at the 0.003 level (Bonferroni corrected)*.

**Figure 1:**
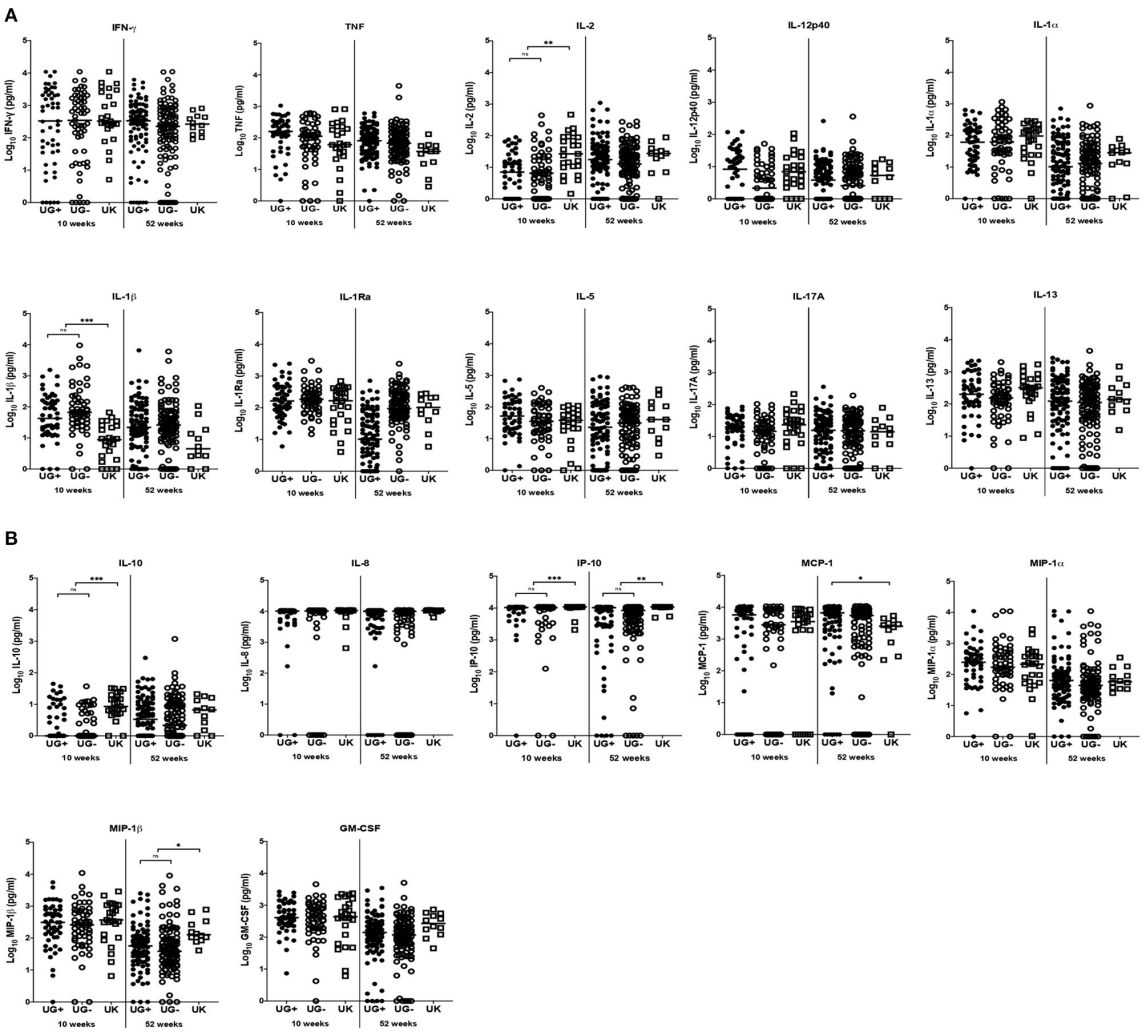
Comparison of immune response to PPD in infants from Uganda and United Kingdom. **(A)** Median IFN-γ, TNF, IL-2, IL-12p40, IL-1α, IL-1β, IL-1Ra, IL-17A, IL-5, and IL-13 production following 6-day stimulation of whole blood with PPD in infant samples obtained at 10 and 52 weeks after BCG immunization. These are presented on the log scale in pg/ml. Closed and open circles represent Ugandan infants of mothers with (UG+) and without (UG–) LTBI, respectively. Open squares represent infants in the UK. The asterisk (*) represents **p < 0.001, ***p < 0.0001. ns, not statistically significant. **(B)** Median IL-10, IL-8, IP-10, MCP-1, MIP-1α, MIP-1β and GM-CSF production following 6-day stimulation of whole blood with PPD in infant samples obtained at 10 and 52 weeks after BCG immunization. These are presented on the log scale in pg/ml. Closed and open circles represent Ugandan infants of mothers with (UG+) and without (UG–) LTBI, respectively. Open squares represent infants in the UK. The asterisk (*) represents **p < 0.001, ***p < 0.0001. ns, not statistically significant.

At week 10, the production of three of the 11 cytokines measured differed significantly between Ugandan and UK infants following stimulation with PPD (Table 2 and Figure 1). Median IL-1β responses were significantly higher in Ugandan infants than in UK infants (*p* < 0.0001; Table 2 and Figure 1A), whereas median IL-2 (*p* = 0.0001) and IL-10 (*p* < 0.0001) responses were significantly higher in the UK compared to Ugandan infants (Table 2 and Figure 1). However, these differences in responses were not sustained at week 52.

At weeks 10 and 52, the production of IP-10 (*p* < 0.0001, *p* = 0.0001, respectively) in response to PPD stimulation of infant blood was significantly higher in UK than in Ugandan infants (Table 2 and Figure 1B). At week 52, production of MIP-1β was significantly higher in UK infants than in Ugandan infants (*p* = 0.0013, Table 2 and Figure 1B).

### Immune Response to ESAT6/CFP10 Stimulation

Cytokine and chemokine responses following stimulation of infant whole blood with a combination of ESAT6 and CFP10 proteins were measured to assess exposure to or infection with *M. tuberculosis* or NTM.

**Figure 2:**
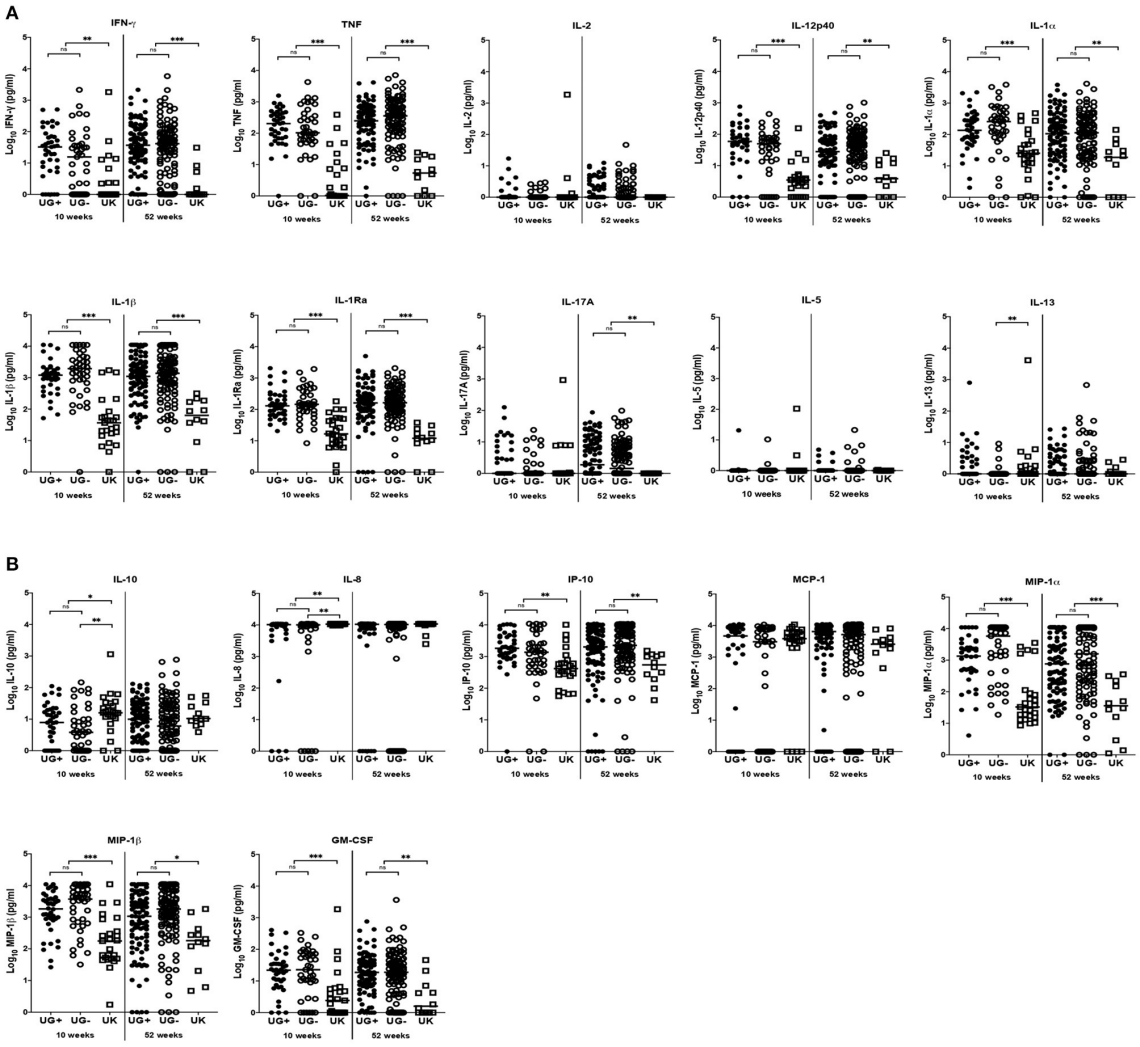
Comparison of immune response to ESAT6/CFP10 in infants from Uganda and United Kingdom. **(A)** Median IFN-γ, TNF, IL-2, IL-12p40, IL-1α, IL-1β, IL-1Ra, IL-17A, IL-5, and IL-13 production following 6-day stimulation of whole blood with ESAT6/CFP10 in infant samples obtained at 10 and 52 weeks after BCG immunization. These are presented on the log scale in pg/ml. Closed and open circles represent Ugandan infants of mothers with (UG+) and without (UG–) LTBI, respectively. Open squares represent infants in the UK. The asterisk (*) represents **p < 0.001, ***p < 0.0001. ns, not statistically significant. **(B)** Median IL-10, IL-8, IP-10, MCP-1, MIP-1α, MIP-1β, and GM-CSF production following 6-day stimulation of whole blood with ESAT6/CFP10 in infant samples obtained at 10 and 52 weeks after BCG immunization. These are presented on the log scale in pg/ml. Closed and open circles represent Ugandan infants of mothers with (UG+) and without (UG–) LTBI, respectively. Open squares represent infants in the UK. The asterisk (*) represents **p < 0.001, ***p < 0.0001. ns, not statistically significant.

At weeks 10 and 52, the production of IFN-γ, TNF, IL-12p40, IL-1α, IL-1β, IL-1Ra, IP-10, MIP-1α, MIP-1β, and GM-CSF were significantly higher in Ugandan than in UK infants following stimulation of blood with ESAT6/CFP10 (Table 3 and Figure 2). At week 10, blood samples from infants in the UK produced significantly higher IL-10 (*p* = 0.0038) and IL-8 (*p* = 0.0005) than Ugandan infants (Table 3 and Figure 2B), although these responses were not sustained at week 52. At week 52, blood samples from Ugandan infants produced significantly higher amounts of IL-17A than UK infants (*p* = 0.0009; Table 3 and Figure 2A).

**Table 3:** Median responses to ESAT6/CFP10 in infants from Uganda and United Kingdom at 10 and 52 weeks following BCG immunization.

**Cytokine/ Chemokine (pg/ml)**	**10 weeks after BCG**			**52 weeks after BCG**		
	**Uganda(*n* = 80)**	**United Kingdom(*n* = 25)**	***p*-value**	**Uganda(*n* = 205)**	**United Kingdom (*n* = 12)**	***p*-value**
IFN-γ	20.64	0.000	**0.0001**	39.49	0.000	**<0.0001**
TNF	135.2	0.04	**<0.0001**	313.2	4.395	**<0.0001**
IL-2	0.000	0.000	0.4912	0.000	0.010	0.1019
IL-12p40	54.14	2.48	**<0.0001**	37.37	2.87	**0.0003**
IL-1α	194.2	24.17	**<0.0001**	107.9	17.74	**0.0007**
IL-1β	1314	35.57	**<0.0001**	1,271	65.39	**<0.0001**
IL-1Ra	134.90	16.03	**<0.0001**	160.80	11.05	**<0.0001**
IL-17A	0.000	0.000	0.2819	0.685	0.000	**0.0009**
IL-5	0.000	0.000	0.5495	0.000	0.000	0.7781
IL-13	0.000	0.000	0.2347	0.000	0.000	0.8188
IL-10	4.200	14.94	**0.0038**	6.960	9.495	0.1451
IL-8	10,112	10,621	**0.0005**	10,404	10,740	0.0739
IP-10	1,587	415.7	**0.0001**	2,064	552.4	**0.0009**
MCP-1	3,169	3821.8	0.8852	6,117	2,552	0.0509
MIP-1α	2,010	31.86	**<0.0001**	936.1	35.61	**<0.0001**
MIP-1β	3,003	176	**<0.0001**	1472	182.0	**0.0012**
GM-CSF	21.59	1.41	**<0.0001**	17.78	0.605	**0.0005**

*P-values are from the Mann Whitney-test for comparison of responses between infants from Uganda and those from the UK. Bold-faced values represent statistically significant p-values at the 0.003 level (Bonferroni corrected)*.

### Comparison of Responses Based on Exposure to Maternal LTBI in Ugandan Infants

We expected that LTBI would be much more common in Ugandan mothers compared to UK. We compared immune responses in infants of mothers with and without LTBI in Uganda with those from UK infants of mothers without LTBI. As previously reported (17), responses to both PPD and ESAT6/CFP10 were remarkably similar between Ugandan infants of mothers with and without LTBI. However, we expected responses in infants of Ugandan mothers without LTBI to be closer to responses from UK infants.

### Immune Response to PPD Stimulation

The responses to PPD stimulation of infant blood were generally similar between infants in Uganda and the UK regardless of maternal LTBI exposure in Ugandan infants, except for MCP-1 responses at week 52 where Ugandan infants of mothers with LTBI had significantly higher responses than UK infants (*p* = 0.0024; Figure 1B).

### Immune Response to ESAT6/CFP10 Stimulation

At week 10, the production of IL-13 (*p* = 0.0020, Figure 2A), IL-10 (*p* = 0.0022, Figure 2B), and IL-8 (*p* = 0.0001, Figure 2B) following stimulation of infant blood with ESAT6/CFP10 was significantly higher in UK infants than in Ugandan infants of mothers without LTBI, but comparable to responses from Ugandan infants of mothers with LTBI. However, these responses were not sustained at week 52.

## Discussion

Based on previous results comparing infants in Malawi and the UK we had expected differences in immune responses to BCG immunization between infants in Uganda and the UK. Immunization of infants in Malawi with BCG has been shown in previous studies to elicit lower IFN-γ responses to PPD than infants in the UK (11, 23) and, in a more comprehensive analysis where more cytokines and chemokines were measured, seven of 42 cytokines and chemokines tested were higher in the UK infants (Th1 responses) while 20 were higher in Malawian infants (innate proinflammatory, regulatory, Th2, and Th17 cytokines and growth factors) at 12 and 52 weeks (13).

We show that infant responses to PPD following BCG immunization were much more similar between Ugandan and UK infants than expected, with no suggestion of suppression of key Th1 responses in Uganda and no Th2 bias as previously observed in Malawi (13). There were a few differences at week 10 but these were not sustained at week 52 so arguably of no long-term significance to protective immunity against TB in the infants. Surprisingly, infants in the UK mounted similar immune responses to Ugandan infants of mothers with LTBI for some of the cytokines and chemokines analyzed.

There were important differences between our study and the Malawi study (13). First, BCG vaccination was administered at birth or within the first week in our study vs. between 3 and 13 weeks in Lalor's study. Differences in immune responses when BCG vaccination is given at birth vs. when delayed have been previously reported (24, 25). Second, there were differences in the assays performed: we used 1/5 dilution of whole blood whereas the Malawi/UK study used 1/10 dilution; we used 10μg/mL of PPD vs. 5μg/mL in Lalor's study. Third, the number of infants analyzed also differed (our Ugandan study sampled more infants than the Malawian study, but similar number of infants were sampled in the UK for both studies). These study-related differences could have contributed to the observed differences between responses in infants in our study and those in the Malawi/UK study. In previous studies in Malawi, infant responses to PPD at early time points were shown to be influenced by geographic location, season of birth and timing of vaccination (11, 14). It is possible that infants in Malawi were vaccinated or blood samples collected after exposure to environmental mycobacteria that are common in the tropics, with the consequent low IFN-γ and high immunoregulatory and Th2 responses observed (26). Factors other than mycobacterial exposure may also explain the Malawi-UK differences. Of note, the Malawi study was conducted in a more rural setting than the Ugandan study and so factors such as nutrition, helminth and malaria infections might be different. Also, the adopted EPI vaccine schedules are different between Uganda, Malawi and the UK. Other differences between our study and the previous studies in Malawi, The Gambia, Indonesia and the UK that could have resulted in the observed differences include the use of BCG-Danish strain in our study (vs. BCG-Russia in the study in The Gambia (14) and attenuated live *M. bovis* strain Paris in Indonesian study (15). Differences in immune responses to different strains of BCG vaccine have previously been reported (27). Previous studies have also attributed differences in responses to vaccines to the genetic background of the individuals (28, 29).

EAST6 and CFP10 are considered to be *M. tuberculosis*-specific antigens (although some species of NTM express them). We measured responses to ESAT6 and CFP10 in order to assess exposure to *M. tuberculosis in utero* and acquisition of responses to *M. tuberculosis* during the first year of life. Responses to ESAT6/CFP10 were generally higher in Ugandan infants for most cytokines and chemokines measured, at both week 10 and 52, indicating a much greater exposure to *M. tuberculosis* (or possibly to ESAT6/CFP10-containing NTM) in Uganda than the UK (26). Moreover, the IFN-γ and TNF responses to ESAT6/CFP10 in infants in Uganda increased with age (17), which would also support progressive exposure over time to or infection with *M. tuberculosis* or NTM. The concentrations of other cytokines and chemokines in response to ESAT6/CFP10 stimulation were overall higher in Ugandan infants than in the UK. The ESAT6/CFP10-expressing NTM such as *M. szulgai* (30) have previously been identified in Ugandan infants and adolescents where the overall prevalence of NTM in infants from rural Uganda investigated for pulmonary tuberculosis was 3.7% (31).

We were interested in whether the differences observed for overall median responses to mycobacterial antigens between Ugandan and UK infants were influenced by maternal LTBI exposure of Ugandan infants, though we recently demonstrated that, in Uganda, maternal LTBI does not influence infant responses to BCG immunization (17). Similarly, in this analysis we did not find differences in the immune response between infants of mothers with and without a LTBI for most cytokines except for IL-10, IL-13, and IL-8 responses to ESAT6/CFP10 stimulation that were higher in UK infants than in Ugandan infants of mothers without LTBI, at 10 weeks. This is a surprising, but interesting result since we expected the infants in the UK and Ugandan infants of mothers without LTBI to mount relatively similar immune responses. However, these responses were not sustained at 52 weeks and therefore may not have long-term significance for protective immunity against TB in the infants. Although statistically significant differences were obtained between the infants of Ugandan mothers with or without LTBI for these cytokines, the values were either very low (IL-13) or very high (IL-8). Thus, differences in cytokines with concentrations in the mid-range such as IL-10 may be more meaningful. IL-8 plays an important role during granuloma formation and is involved in recruiting neutrophils, T cells and monocytes to the site of infection (32, 33). Production of IL-8 by neonatal T cells has also recently been demonstrated as an anti-microbial effector mechanism (34). Th2 immunity, characterized by IL-4 and IL-13, is thought to antagonize protective Th1 responses to TB (35). Th2 activation of macrophages results in alternatively activated phenotypes that are less potent in their antimicrobial activity (36). IL-10 is an immunomodulatory cytokine with a potential effect on many cells, including macrophages (37). IL-10 has been shown to regulate phagosome maturation during human *M. tuberculosis* infection thus highlighting its potential role in sustaining pathogen persistence (38). Collectively, the fact that these cytokines were present in greater concentrations in the UK infants (although with the caveats regarding concentrations discussed above) could imply either beneficial or detrimental effects on anti-mycobacterial immunity.

Contrary to the above observations, median MCP-1 response to PPD stimulation was significantly higher in Ugandan infants of mothers with LTBI than in UK infants, at week 52. MCP-1 is thought to contribute to the inflammatory responses driven by T lymphocytes (39, 40). This may partly explain the lack of differences in the Th1 or proinflammatory responses observed between infants in Uganda and the UK.

Additional assays such as intracellular cytokine staining and flow cytometry would be needed to identify the cellular sources of these cytokines and chemokines. We chose the Luminex assay instead as our primary read out, so that we could assess a range of cytokine/chemokine responses. Moreover, cell phenotype and function following BCG immunization of infants have previously been assessed by our team and others (9, 41–43).

The finding that there is no difference in Th1 responses to PPD between infants in Uganda and the UK calls for a re-evaluation of our understanding of the variability in protection afforded by BCG. In the UK, only infants of mothers without LTBI were included. The remarkable similarity in IFN-γ responses to PPD between Ugandan and UK infants post-BCG may suggest that the stimulus of BCG was able to override effects of prior (prenatal), or even concurrent, mycobacterial exposures (or lack of them).

Previous studies in Uganda and the UK assessed innate immune responses in cord blood and in BCG vaccinated infants, measuring cytokine responses after 24 h of stimulation (16, 44), although the design of the Ugandan study did not allow assessment of post-vaccination immune responses. Our recent longitudinal study (17) showed very high proportions of infants who produced high cord blood cytokine levels in response to stimulation with mycobacterial antigens, regardless of mother's TB exposure status. However, there was no evidence of differences in the subsequent evolution of responses to PPD based on cord blood cytokine profiles. In the present study a longer 6-day culture period was used to enable restimulation of antigen-specific memory T cell responses, so although we cannot rule out that these may have been influenced by earlier release of innate cytokines, or that some cytokines derived from innate immune cells might still be present [as a result of influence of innate immunity on adaptive immunity (45) and BCG-induced increases in function of innate cells (46, 47)], we predict that most of the cytokines are derived from T cells.

One limitation of recruiting volunteers in the UK was that the study period overlapped with a global BCG shortage that also affected the UK (48), thus restricting the number of BCG vaccinated infants that could be recruited within the study period. Moreover, the smaller than planned sample size did not impact the findings, since we see differences for ESAT6 alongside no differences for PPD. Another limitation of the study is the small amount of blood available from infants and this limited the number of assays possible. This is a common occurrence with exploratory immunological studies. Moreover, we used an assay (Luminex) that enabled us to assess a range of cytokines/chemokines using the available amount of sample (which was sufficient for this purpose). We were therefore able to address the objectives of the study. In the UK, by study design, infant samples were only collected at weeks 10 and 52 and from infants of mothers without LTBI. Thus, we did not have pre-vaccination or cord blood samples collected from UK infants for a pre-vaccination comparison with the infants in Uganda. As TB incidence is relatively low in the UK, recruitment of mothers with LTBI would require a targeted recruitment drive (for example, identifying contacts of TB cases) which this study was not designed to do. Also, we did not adjust for confounders since the data available was quite minimal.

In conclusion, immune responses to mycobacterial antigens following BCG immunization are similar for PPD, but differ for ESAT6/CFP10, between infants in Uganda and the UK. We show that neither maternal LTBI nor infant exposure to or infection with *M. tuberculosis* (or NTM) impacts response to PPD following BCG immunization. The observed differences in immune response to, and efficacy of, BCG immunization are likely to be due to other causes.

## Data Availability Statement

The raw data supporting the conclusions of this article will be made available by the authors, without undue reservation.

## Ethics Statement

The studies involving human participants were reviewed and approved by Uganda Virus Research Institute Research Ethics Committee (Reference GC/127/13/09/16 and GC/127/16/03/434), Uganda National Council for Science and Technology (Reference HS 1526) and London School of Hygiene and Tropical Medicine (Reference 7104 for the Uganda study, 8720-1 for the UK study) and 15/LO/0048 for the NHS in the UK study. Written informed consent to participate in this study was provided by the participants' legal guardian/next of kin.

## Author Contributions

AE, SC, PM, PK, EW, SS, and HD conceived the study and secured funding. PM, GN, MN, and MH-A performed all laboratory assays. RB, LD, and AG were members of the clinical team involved in recruitment, follow up, and clinical reporting in the UK. AE, SC, SS, HD, EW, LL, and PM reviewed the data and wrote the initial drafts of the manuscript. LL and EW provided statistical expertise and support. All authors read and approved the final version of the manuscript.

## Conflict of Interest

The authors declare that the research was conducted in the absence of any commercial or financial relationships that could be construed as a potential conflict of interest.
